# Ambient climate and experimental drought strongly shape the richness and abundance of soil microbial communities in a tropical montane cloud forest

**DOI:** 10.1093/femsec/fiag112

**Published:** 2026-10-12

**Authors:** Sarah J Sapsford, Patrick H Martin

**Affiliations:** Harry Butler Institute, Murdoch University, Western Australia, Murdoch 6150, Australia; Department of Biological Sciences, University of Denver, Denver, CO 80208, United States

**Keywords:** bacteria, decomposition, elevation, fungi, microbial community composition, soil properties

## Abstract

Tropical montane forests are undergoing rapid climate change, with declining cloudiness and warming in higher elevations. While impacts on vegetation are increasingly documented, effects on belowground microbial communities and ecosystem processes remain poorly studied. We investigated how experimental drought and warming influence soil microbial communities and decomposition across a 900-m elevation gradient in the tropical montane forests of the Dominican Republic. Precipitation exclusion shelters simulated drought at high (2250 m a.s.l.) and low elevation (1350 m a.s.l.) and high elevation soils were translocated to the lower elevation to simulate warming. Fungal and bacterial communities were measured by DNA sequencing over 11 months, in addition to soil properties and litter decomposition. Fungal richness increased under drought but was unchanged following translocation, whereas bacterial richness and abundance were largely unaffected by either treatment. Decomposition was consistently faster at the lower (warmer) site but slowed under intense drought at both elevations. Soil carbon was higher at high elevation, consistent with slower decomposition. Overall, these results indicate cooler and wetter conditions promote structurally and functionally complex fungal communities. As temperature and precipitation patterns change, fungal communities are more likely to shift in response, altering decomposition and nutrient cycling, with implications for carbon storage in tropical montane ecosystems, especially at high elevations.

## Teaser

Experimental drought and warming across a tropical montane elevation gradient revealed contrasting belowground responses. Drought increased fungal richness and reduced litter decomposition, while bacterial communities were largely unaffected by either treatment. Decomposition was lower at low elevation, highlighting how changing temperature and precipitation regimes may influence microbial communities and carbon cycling in tropical montane forests.

## Introduction

The climate on tropical mountains changes rapidly over short distances, with cloud, temperature and precipitation patterns varying markedly with elevation (Giambelluca and Nullet 1991, Bruijnzeel et al. 2011, Martin and Fahey 2014). These fluctuating conditions play a central role in the ecological patterns of the vegetation community, leading to striking changes in composition, richness and physiognomy of the vegetation along the elevation gradient (Sherman et al. 2005, Martin et al. 2007, Bruijnzeel et al. 2011, Fahey et al. 2016). In contrast, the distribution and functioning of soil microbes on tropical elevation gradients is less clear (Dalling et al. 2016, Martin and Bellingham 2016), as high-throughput sequencing of microbial communities in tropical montane forests remains comparatively uncommon (e.g. Peay et al. 2017). This knowledge gap is important because soil bacteria and fungi regulate ecosystem processes including nutrient cycling (e.g. Che and Jin 2024), organic matter decomposition (e.g. Schneider et al. 2012) and carbon storage (e.g. Averill and Hawkes 2016), with feedbacks on the surrounding vegetation.

Environmental conditions at higher elevations in tropical montane forests undoubtedly shape microbial communities in distinctive ways (Davidson and Janssens 2006). Lower temperatures, higher precipitation and frequently waterlogged and anoxic soils on tropical mountains (especially in the cloud zone; Silver et al. 1999, Roman et al. 2010, Fahey et al. 2016) alter microbial physiology, slow rates of decomposition (Vitousek et al. 1994, Silver et al. 1999, Schuur et al. 2001, Dalling et al. 2016, Ostertag et al. 2022) and lead to the buildup of soil organic matter (SOM) (Girardin et al. 2010, Dieleman et al. 2013). As a result, SOM typically peaks at higher elevations in the tropics (Tanner et al. 1998), and the quantity and quality SOM in turn exerts a strong influence on microbial composition and abundance (Orwin et al. 2006, Whitaker et al. 2014, Peay et al. 2017), suggesting that microbial communities may exhibit distinct elevational patterns that differ from plants. Indeed, these environmental patterns provide strong mechanistic basis to expect shifts in microbial communities and ecosystem functioning along elevation gradients, yet relatively few studies have directly examined how these interacting changes in climate and soil organic matter influence soil microbial communities across tropical montane forests.

Understanding the relationships of soil microbial communities across tropical elevational gradients has become increasingly important because tropical montane ecosystems are experiencing accelerated climate change (Williams et al. 2007, Laurance et al. 2011, Krishnaswamy et al. 2014, Seddon et al. 2016, Hannah 2021) and weather extremes, especially droughts (Foster 2001, Scholl et al. 2021, Swann et al. 2023). Upper montane elevations in the tropics are warming substantially faster than lower elevations (Pepin et al. 2025) and systematic changes in climate are causing orographic cloud patterns to shift on tropical mountains, as the elevation of the cloud base increases due to a rise in sea surface temperatures (Loope and Giambelluca 1998, Lawton et al. 2001). As clouds move higher on tropical mountains (Still et al. 1999, Karmalkar et al. 2011), formerly cloudy areas become markedly hotter and drier, resulting in plant and animal migrations (Colwell et al. 2008, Feeley and Silman 2010, Laurance et al. 2011, Wiens 2016, Muñoz et al. 2022). Given the sensitivity of microbes to changes in moisture (Hawkes et al. 2011, Evans and Wallenstein 2012, Evans et al. 2022), warmer temperatures, increased droughts and reduced cloudiness are likely to fundamentally alter soil microbial communities in tropical montane ecosystems as well. However, the responses to microbial communities to these climatic shifts remain unknown.

Tropical mountains provide natural space-for-time settings to assess how future climate change may influence the structure and function of biota and ecosystem processes (Malhi et al. 2010). The microbial community in warmer and drier areas at lower elevations, particularly in areas below the cloud belt, can be informative of future changes coming to the microbial community in the cooler and wetter upper montane cloud forest (Peay et al. 2017). Similarly, soil transplants on the elevation gradient can simulate future changes in the microbial community, like compositional shifts under increasingly warmer and drier conditions (Glassman et al. 2018); such studies have found that soil respiration (Zimmermann et al. 2009) and rates of litter decomposition (Scowcroft et al. 2000, Salinas et al. 2011) are higher at lower elevations. If decomposition increases widely with such changes, climate change could shift tropical montane forests from a carbon sink to a source (Biskaborn et al. 2019). However, few studies have combined natural elevation gradients with experimental climate manipulations to determine how soil microbial communities and their associated ecosystem functions respond to warming and drought across tropical montane forests. While climate patterns are undoubtedly paramount in structuring tropical montane ecosystems, natural disturbances can be common and increase turnover and dynamics in these systems (Crausbay et al. 2015, Crausbay and Martin 2016).

In this study, we asked how the microbial community is structured by changing environmental conditions along the elevation gradient in the tropical mountains of the Cordillera Central, Dominican Republic. The Cordillera Central is an important ecosystem, containing one of the largest areas of montane forests in the Caribbean (Gannon and Martin 2014), much of which was protected in national parks established before any significant clearing or land use had occurred in the region (Martin et al. 2007), providing an unusually pristine site for studying the microbial community. In particular, we asked how the abundance, richness and functional type of the microbial community in the lower montane forest differs from the microbial community in the upper montane forest located in the cloud zone. By experimentally simulating higher temperatures (with soil transplants), drought (with rain shelters) and decomposition (with litterbags), we sought to determine how patterns and processes in the microbial (bacteria and fungi) community are structured by these contrasting climates.

Due to the cooler, wetter and more anoxic conditions in the upper montane cloud forest, with concomitantly slower decomposition and higher organic matter accumulation, we expected 1) the upper montane forest to support lower microbial richness. We hypothesized 2) the soil microbial community in the upper montane cloud forest would be more sensitive to environmental change because they are adapted to persist under consistently cool and moist conditions. Consequently, under experimental drought, we expected 3) decomposition rates to increase faster in the upper montane cloud forest as anoxic conditions are reduced. We also expected 4) experimental drought to decrease microbial richness in both elevations due to their sensitivity to moisture, but to cause larger shifts in the microbial community of the upper montane cloud forest.

## Methods

### Site description

The microbial community was studied in a lower montane site (1350 m a.s.l.) and an upper montane site (2250 m a.s.l.) in tropical montane forests on the windward slopes of the Cordillera Central in Armando Bermudez National Park, Dominican Republic (19.035°N, 70.910°W). Both sites are in pristine forest stands, with no history of direct human impacts (logging, grazing, etc.). Situated below the orographic cloud zone, the lower site is classified as lower montane tropical broadleaf forest and the upper site—in an area with frequent orographic cloud (> 75% of days/year; Martin and Fahey 2014)—as tropical montane cloud forest (Sherman et al. 2005). The soils in the sites have not been formally mapped or classified, but a standard laboratory analysis of surface soil samples (the top 15 cm) reported the lower site has a sandy clay loam texture, a pH of 5.2, and low total soil carbon (8.2%) and nitrogen (0.7%), and the upper site has a loamy fine sand texture, a pH of 5.1, and markedly higher total soil carbon (39.6%) and nitrogen (2.8%) (Martin et al., unpublished data).

The forest in the lower site has a canopy 25–28 m tall and is dominated by the tree species *Nectandra coriacea, Ocotea leucoxylon, Alchornea latifolia*, and a palm, *Prestoea acuminata* var. *montana* (Martin et al. 2004, 2011). The cloud forest, where the canopy is lower (15–18 m) and epiphytic bryophytes are abundant, is dominated by tree ferns (*Cyathea* and *Alsophila* spp.) and the tree species *Podocarpus buchii* and *Antirhea oligantha* with patches of *Brunellia comocladifolia* (Martin et al. 2007). Above-ground biomass and tree growth rates decline markedly with elevation in the site (Sherman et al. 2012). Mean annual air temperature is 18°C at the lower and 13°C at the upper site, and the sites have similar mean annual precipitation of 1800–1900 mm (Martin and Fahey 2014). A dry season typically occurs each year from January–March across the region, but it is much drier (45% less precipitation on average) at the lower site than the upper site (Martin and Fahey 2014). While the Cordillera Central is impacted regularly by cyclones (Gannon and Martin 2014, Van Bloem and Martin 2021, Ibanez et al. 2022) and wildfires (Martin and Fahey 2006, Sherman et al. 2008, Swann et al. 2023, Swann et al. 2024), both sites had closed canopies and no evidence of any recent disturbances before and during the study.

### Field sampling and drought treatments

The soil microbial community was sampled in each site 3 times in 2018: pre-treatment in mid-January, mid-treatment in mid-June, and final-treatment in mid-December. In each site, 24 replicate plots (1 × 1 m) were established in the understory, with 12 control plots (measuring ambient conditions) and 12 treatment (drought) plots. Control and treatment plots were placed in pairs with a gap of 1.5 m between pairs to prevent impacts between the plots. Pairs of plots were placed randomly in each site but confined to areas with gentler slopes (∼10–15°) to minimize lateral subsurface water flow, and to prevent impacts from water draining downslope from the drought shelters, pairs were placed laterally along to the slope such that no pairs or plots were located up- or down-slope of other plots. Each pair of plots was located >10 m from the nearest neighbouring pair, resulting in a total area of ∼0.1 ha per site.

Experimental drought was imposed via precipitation exclusion with rain deflection shelters. Using validated designs (Drought-Net 2018), shelters were made with 1 m^2^ panels of clear polycarbonate roofing (Suntuf, Kutztown, PA, USA) mounted 1 m above the forest floor on a PVC frame and angled to direct rainwater downslope away from the plots (Fig. S1). Such shelters effectively reduce or eliminate precipitation without significant effects on soil and air temperatures and light levels (e.g. Heisler-White et al. 2008, Cherwin and Knapp 2012, Goke and Martin 2022, 2023).

To explore how different types of drought impact the microbial community, we used 3 drought treatments, which we termed ‘chronic’ (50% reduction in precipitation for 11 months, 6 replicates per site), ‘long intense’ (100% reduction for 11 months, 3 replicates per site), and ‘short intense’ (100% reduction for 5 months, 3 replicates per site). Shelters with 50% open area were used for the ‘chronic’ treatment; 0% open area for the ‘long intense’ treatment; and 0% open area for the ‘short intense’ treatment with its shelters removed in mid-June, simulating a drought ending to explore the capacity of the microbial community to recover from a deep but short drought. We expected the effect size in the 100% drought treatments (‘long intense’ and ‘short intense’) to be large, thus requiring fewer replicates (n = 3) than the milder ‘chronic’ treatment (n = 6).

Soils were collected in all 48 plots at each sampling date. Loose leaf litter was removed and the topsoil was collected with a volumetric soil corer (7.5 cm diameter × 12.5 cm depth). After collection, the soil samples were air dried, sieved once dry with a 2 mm sieve to remove any coarse fraction, and homogenized with a ball mill grinder in preparation for chemical analysis at the Dalling lab (University of Illinois) for soil pH, and total C and N as a percentage of weight (see Baldeck et al. 2013 for detailed laboratory methods). In the drought treatments, the sample core was collected directly under the center of the drought shelter, away from the shelter edge. A subsample of each core was used for fungal and bacterial analyses per below. Soil moisture was measured for each soil sample (144 in total) using gravimetric (oven-drying) methods. At each sampling date, soil temperature was measured in all plots with a TempPro APT418 dial analog thermometer with a 12.7 cm probe inserted into the ground; this thermometer averages the temperature along the probe from 6 cm to 12 cm depth. Soil temperature and moisture (volumetric water content) were also recorded every 15 min through the duration of the study in a subset of plots (3 per treatment per site) with two types of probes, EC-5 (soil moisture only) and 5TM (soil moisture and temperature), attached to EM50 dataloggers (Decagon Devices, Pullman, WA, USA); probes were placed horizontally 7.5 cm below the soil surface in the center of a plot.

### Microbial community translocation experiment

We used a translocation experiment with microbial ‘cages’—which have been effective in isolating microbial communities in other studies (e.g. Allison et al. 2013)—to simulate warmer temperatures on the cloud forest microbial community. Soil was collected with the corer in ambient conditions at the upper site in January 2018, gently homogenized by hand, and subsampled (8 g) to place in a microbial cage. Each cage was 10 cm × 10 cm x 1.5 cm in size and made of nylon mesh with a pore size of 0.2 μm (Maine Manufacturing, ME, USA), small enough to prevent outside microbes from colonizing the cage, while still allowing abiotic exchange (e.g. water) with the environment. Microbial cages are mesh envelopes or pouches, which are flat, white, and square in appearance, and are sealed along the edges with a ‘hot-melt’ adhesive applicator.

Cages were placed on the forest floor in the ambient, chronic and long-intense drought treatments in both sites (4 replicates per treatment, 24 cages in total for each time point) and lightly covered with extant leaf litter to mimic natural conditions. The cages were collected after 5 months and 11 months, stored at −20°C, and transported to the University of Denver for analysis. Only 3 replicates per treatment (36 cages in total across both time points) were successfully sequenced for fungi (see methods below) and 42 cages in total were sequenced for bacteria, but the treatments remained balanced.

### Decomposition

In mid-January 2018, we deployed 96 litterbags in each site: 48 in ambient (control) conditions, 24 in ‘chronic’ drought, 12 in ‘long intense’ drought, and 12 in ‘short intense’ drought treatments. Half the litterbags were collected after 5 months and the remainder after 11 months. The litterbags were prepared before deployment using leaf material from a tropical montane tree species, *Podocarpus gracilior* (a congener of a dominant species in our study site, *P. buchii*), acquired from the Denver Botanic Gardens. Whole leaves were dried at 70°C to a constant mass and placed in nylon mesh bags (10 × 15 cm, 1000-μm mesh; Component Supply Company, Fort Meade, FL) with an initial mass of 1.00 ± 0.01 g (mean ± 1 SD). A 1000-μm mesh was used to exclude only macrofauna (Gongalsky 2021) from decomposition rates. After retrieval, the remaining leaf contents were gently washed with tap water, and any soil or root material attached to the litter was removed with tweezers; the samples were then dried to a constant mass at 70°C and weighed to ± 0.01 g.

### Fungal and bacterial abundance

Bacterial and fungal abundance were measured using methods in Frey et al. (1999) using epifluorescence staining. Fungal hyphal length was measured with standard techniques (Brundrett et al. 1996) as a metric of total soil fungal abundance; while standard hyphal extraction methods can have limits in highly organic soils (Camenzind and Rillig 2013), our standard method across sites and treatments allows for a direct comparison of the relative number of fungal hyphal lengths between samples in a given amount of soil. Briefly, to collect hyphae, a random subsample (4 g wet weight) of each soil sample collected in the study was extracted with a 1.5 M solution of sodium hexametaphosphate and filtered through 0.2 μm nylon mesh. Filters were stained (acid fuchsin) (Frey et al. 1999), mounted on a glass slide with a medium (polyvinyl lactic acid), and dried at 65°C overnight. Hyphal lengths were measured with a gridline intersect method at 200× on a Nikon phase-contrast microscope (Nikon Eclipse e400, Lake Forest, CA, USA).

For bacterial abundance measurements, a random subsample (1 g dry weight) of each soil sample collected in the study was extracted and placed on microscope slides for cell counts of bacteria; abundance values were calculated as cell counts per mg carbon per soil subsample. Quantifying bacteria abundance per mg of carbon from a fixed weight of soil corrects for variation in mineral and organic matter between soil samples, providing a more direct measure of microbial concentration relative to the available organic resource pool.

### DNA extractions

We extracted soil DNA from each sample with the PowerSoil DNA Isolation kit (MoBio, Carlsbad, CA, USA) per manufacturer guidelines. We amplified a ∼340 bp region of the fungal ITS2 gene using a staggered primer design. This included a forward primer (ITS9f; AATGATACGGCGACCACCGAGATCTACAC TC TTTCCCTACA CGACGCTCTTCCGATCT NNNNNGAACGCAGCRAAIIGYGA) and barcoded, reverse primers with the reverse complement of the 3′ Illumina adapter (CAAGCAGAAGACGGCATACGAGAT), a unique 12 base barcode, a pad (AGTCAGTCAG), a linker sequence (CC), and the ITS4 primer (TCCTCCGCTTATTGATATGC). We used a staggered primer design that included 0, 1, 2 or 3 bases preceding the ITS4 primer (e.g. CC-ITS4, CC-G-ITS4, CC-AG-ITS4, or CC-CAG-ITS4; White et al. 1990). This design increases the diversity of amplicon sequences across the Illumina Miseq flowcell early in the read (Tremblay et al. 2015). This improves amplicon detection and sequence quality.

The bacterial amplicon library was prepared using bacterial specific primers 515F (5′-GTGYCA GCM GCC GCG GTA A-3′) and 806R (5′-GGA CTA CNV GGG TWT CTA AT-3′) which amplify the 16S V4 region (Caporaso et al. 2012). DNA quality and concentrations were quantified using a NanoDrop and standardized to 10 ng/μl prior to PCR amplification.

Each PCR reaction included: 21.5 μl of Platinum PCR Supermix (Invitrogen, Carlsbad, CA), 0.75 μl of each primer (10 μM), 1 μl of BSA (10 mg mL^−1^), and 1 μl of DNA (10 ng). Reactions ran with a hot start at 94°C for 5 min, 35 cycles of 95°C for 45 s, 50°C for 1 min, 72°C for 90 s, and a final extension step of 72°C for 10 min. PCR reactions were ran in triplicate, pooled, and purified with Agencourt AMPure XP magnetic beads (Beckman Coulter, Brea, CA). We quantified purified samples using the Qubit dsDNA High Sensitivity Assay Kit (Life Technologies, Grand Island, NY).

Samples were sequenced as 2 × 300 bp paired end reads on one lane of an Illumina MiSeq sequencer. Sequencing was performed at the Genomics core in the Institute for the Integrative Genome Biology at the University of California, Riverside.

### Bioinformatics

Paired-end reads (with adaptor sequence removed) were merged using USEARCH v11 (Edgar 2010) allowing a maximum of five mismatches between paired reads. Sequence quality filtering and dereplication were carried out using VSEARCH v2.15.2 (https://github.com/torognes/vsearch). Specifically, merged reads were quality filtered based on the maximum expected error rate (maxee = 1), and sequences <200 bp were removed. Quality-filtered sequences were subsequently dereplicated using full-length sequence identity, retaining sequence abundance information. Operational taxonomic units (OTUs) were generated from the dereplicated sequences using the UPARSE cluster_otus algorithm implemented in USEARCH. The original merged reads were then mapped to the representative OTU sequences using the VSEARCH usearch_global algorithm at a minimum sequence identity of 97% to generate the OTU abundance matrix. Fungal OTUs were identified by searching against the public database provided by UNITE v8.3 (10.05.2021) (Abarenkov et al. 2010, Nilsson et al. 2018). Bacterial OTUs were identified by searching against the publicly available SILVA 138.1 database (Quast et al. 2012). After quality filtering, the mean library size for fungi was 21530.39 reads per sample with 1946 OTUs and for bacteria was 44994.35 reads per sample with 4365 OTUs. For the microbial translocation data, the mean library size for fungi was 21530.39 per sample with 944 OTUs and for bacteria was 56581.95 per sample with 3475 OTUs.

Ecological information of each fungal OTU was based primarily on FUNGuild (https://github.com/UMNFuN/FUNGuild), following the user’s manual where assignments were made as ‘probable’ and ‘highly probable’ (Nguyen et al. 2016). Where there was no match or an OTU was ranked as ‘possible’, literature searches were conducted and OTUs assigned to a guild based on published studies. As it is difficult to assign function to fungi found in soil, assignments were conservative. Thus, if more than one guild was applicable to an OTU or if no information was found, we classified these unknown OTUs in an ‘undefined fungus’ guild.

## Data analysis

### Soil properties

All analysis was completed in R version 4.5.1 (R Core Team 2025). We used linear mixed models using the *lmer* function in the package lme4 to test the effects of the three-way interaction of site (upper and lower elevation), treatment (i.e. 4 levels: ambient, chronic, short intense, and long intense) and time (i.e. 0, 5, and 11 months) on each soil property (e.g. soil temperature, pH, carbon, nitrogen, gravimetric moisture, and decomposition). As the Decagon probes only covered a subset of the plots and a number of the probes failed during the study (see Figs S2 and S3), we opted to use gravimetric soil moisture and soil temperature measurements for the statistical analysis, as these data were complete (i.e. no missing values) and were collected at the same time the microbial community was sampled. To incorporate the paired plot design and account for this dependency, plot within pair was used as a random intercept. We first tested a three-way interaction among the variables and retained significant variables. We used the *emmeans* package (Lenth 2020) to calculate estimated marginal means and to conduct post-hoc tests. Chi-squares and *P*-values were calculated using the Anova function in the package car (Fox and Weisberg 2019). Assumptions were verified by visually inspecting residuals for normality and homoscedasticity (Zuur and Ieno 2016). Inspection of residuals indicated heterogeneity of variance among sites with the variable gravimetric soil moisture. To account for this, we refitted the model using the lme function from the nlme package, incorporating a site-specific variance structure (varIdent).

### Microbial abundance and richness

We used linear mixed models in the *lmer* function in the package lme4 to determine patterns in fungal and bacterial abundance and richness across site, treatment, and time (framework as above). As both fungal and bacterial abundance data were right skewed, the data was log-transformed to meet the assumption of normality. To determine fungal and bacterial species richness, OTUs were rarefied based on the minimum number of sequences observed per sample and rarefaction was independently repeated 900 times using the *rrarefy* function in the vegan package (Oksanen et al. 2022). Mean OTU richness across these 900 rarefactions was used in subsequent analyses. Following removal of one fungal sample with extremely low sequencing depth (69 reads), fungal samples were rarefied to 779 reads, bacteria 781 (removal of one sample of 331 reads) in the drought data. With the microbial cage data, fungi were rarefied to 11 784 (removal of one sample of 284 reads) and bacteria rarefied to 23942. Inspection of residuals indicated heterogeneity of variance across time with the bacterial rarefied richness. To account for this, we refitted the model using the lme function from the nlme package, incorporating a time-specific variance structure (varIdent).

In the microbial cage data, plots were not paired, so only plot was used as a random effect to account for repeated measures and otherwise the framework was as described above.

### Microbial community composition

We used the Bray–Curtis dissimilarity index to calculate dissimilarly values from fungal or bacterial OTU relative abundance data to determine differences in the community composition of soil fungi or bacteria as a function of site, time and drought treatment. Bray–Curtis dissimilarity values were calculated with the *vegdist* function in the vegan package (Oksanen et al. 2022). To test for community differences across time, site and drought treatment and interactions, permutational multivariate analysis of variance (PERMANOVA) with 9999 permutations using the function *adonis2* was used in package vegan (Oksanen et al. 2022). Plots were included as a variable for ‘strata’ to control for dependency of plots repeatedly sampled over time. The null hypothesis of a PERMANOVA is the centroids of the groups are equivalent for all groups. Results were visually analyzed in a principle coordinates analysis made with the *pcoa* function in package ape (Popescu et al. 2012).

For fungal functional analysis, the proportion of reads in each functional groups (symbionts (arbuscular mycorrhizal fungi, ectomycorrhizal fungi, orchid mycorrhizal fungi, and ericoid mycorrhizal fungi), saprotrophs, endophytes, and plant pathogens were modelled as a function of site, time, and drought treatment (three-way interaction) using the package and function *glmmTMB* with family betabinomial (Brooks et al. 2017) or a negative binomial if assumptions of the model did not fit using a betabinomial. Best fit model and assumptions were followed as the framework described above. Fungi classified as ‘other’ (i.e. animal pathogens, biotrophs, fungal parasites and lichenized fungi) or as ‘undefined’ (no function could be accurately described) were not included in the analysis as they were not the focus in our hypotheses.

## Results

### Soil properties and decomposition

Soil temperature, measured in January (sampling month 0), June (month 5), and December (month11) with an analog thermometer, was consistently higher at the lower montane site (overall mean = 16.4°C ± 0.12 SE), peaked seasonally in mid-June (mean of 17.5°C ± 0.10 SE), and declined in December (mean of 15.8°C ± 0.04 SE). In contrast, temperature at the upper montane site (overall mean = 12.3°C ± 0.15 SE) increased over the sampling period (Fig. 1A), reaching a mean of 13.6°C (±0.06 SE) in December. This diverging pattern is indicative of the significant interaction between site and time for soil temperature (X^2^ (2) = 227.43, *P* < 0.001) but did not differ among treatments (marginal R^2^: 0.949, conditional R^2^: 0.957; Fig. 1A). Over the duration of the study, mean daily soil temperature recorded with Decagon datalogging probes averaged 17.3°C (min 14.3°C; max 18.9°C) in the low elevation site and 12.2°C (min 8.8°C; max 14.8°C) in the high elevation site (Fig. S2).

**Figure 1.**
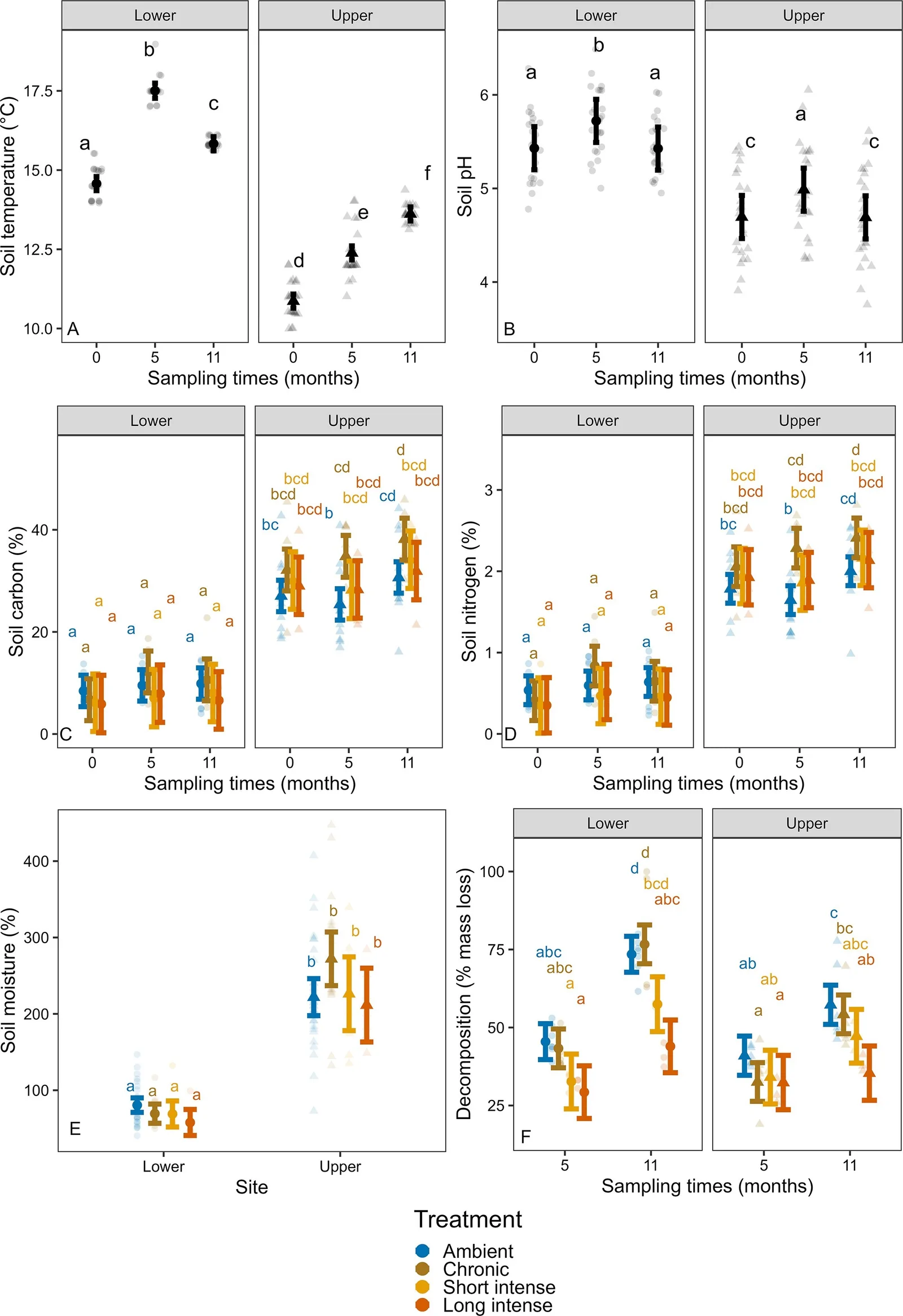
Estimated marginal means (± 95% CI) of (A) soil temperature (°C), (B) soil pH, (C) soil carbon (%), (D) soil nitrogen (%), (E) soil moisture (%), and (F) litter decomposition (% dry mass lost after 5 and 11 months post-deployment) in a lower and upper tropical montane forest over time. Patterns of the four drought treatments—ambient (i.e. control), chronic (∼50% less precipitation for 11 months), short intense (∼100% less precipitation for 5 months), long intense (∼100% less precipitation for 11 months)—are only displayed when there was a significant effect.

Soil pH significantly differed between site (X^2^ (1) = 23.52, *P* < 0.001) and time (X^2^ (2) = 60.72, *P* < 0.001; interaction term not significant), but not among drought treatments (marginal R^2^: 0.456, conditional R^2^: 0.870; Fig. 1B). Soil pH was more acidic at the upper montane site but had a seasonal trend at both sites where pH was highest at the 5 months sampling period (mid-June; 5.0 ± 0.11 at higher site vs 5.7 ± 0.07 at lower site).

Soil carbon was significantly higher at the upper montane site, averaging 27.7% (± 1.24 SE) in ambient plots vs. 9.28% (± 0.50 SE) in the lower montane site (Fig. 1C) and differed over time (site x time interaction: X^2^ (2) = 10.14, *P* = 0.006) and drought treatment (site x drought treatment interaction: X^2^ (3) = 9.85, *P* = 0.020; marginal R^2^: 0.801, conditional R^2^: 0.907). The ambient plots at the upper montane site had significantly lower soil carbon than chronic drought plots at the 5 months sampling period (mid-June). There were no significant posthoc pairwise differences among treatments at the lower montane site.

Soil nitrogen was also significantly higher at the upper montane site (Fig. 1D), averaging 0.59 (± 0.04 SE) in lower montane ambient plots and 1.81 (± 0.07 SE) in upper montane ambient plots, and differed over time (site x time interaction: X^2^ (2) = 10.14, *P* = 0.006) and drought treatment (site x drought treatment interaction: X^2^ (3) = 8.99, *P* = 0.029; marginal R^2^: 0.835, conditional R^2^: 0.906). C: N did not significantly differ across time, treatment or between sites.

Soil moisture (gravimetric) was, on average, 2 times higher at the upper montane site than the lower montane site (X^2^ (1) = 284.96, *P* < 0.001) (marginal R^2^: 0.599, conditional R^2^: 0.611; Fig. 1E) and did not change over sampling period but did differ among drought treatment (site x drought treatment interaction: X^2^ (3) = 8.81, *P* = 0.032); however, pairwise comparisons within site were not significant. Decagon probe data indicated the drought treatments were effective in reducing soil moisture as intended, as volumetric soil moisture averaged 17% in the intense drought treatments, 23% in the chronic drought treatments, and 49% in the ambient (control) treatments (Fig. S3).

Decomposition was significantly faster at the lower montane site, especially after 11 months (site x time interaction: X^2^ (1) = 10.42, *P* = 0.001) and differed among drought treatments over time (time x drought treatment interaction: (X^2^ (3) = 11.92, *P* = 0.008; marginal R^2^: 0.769, conditional R^2^: 0.788; Fig. 1F). In ambient plots at the lower montane site, on average decomposition increased 1.6 fold from month 5 to 11. In comparison, at the upper montane site, decomposition increased 1.4 fold from month 5 to 11. At both sites, there was a significant change in chronic plots over time (1.9 fold at lower and 1.5 fold at upper). Decomposition significantly changed under short intense plots at the lower montane site (1.74 fold from month 5 to 11), but not at the upper montane site (1.40 fold from month 5 to 11). These results show decomposition slows significantly under short and long intense droughts. However, by 11 months, cumulative decomposition in the ambient, chronic and short intense treatments were indistinguishable in both sites, with the main significant difference confined to the long intense treatment plots.

### Soil fungal richness, abundance, and composition

In general, fungal richness trended higher on average in ambient plots at the upper (114.30 ± 3.46) vs lower site (92.57 ± 3.69). The interaction between time and site (X^2^ (2) = 8.47, *P* = 0.015) and time and drought treatment were significant for fungal richness (X^2^ (6) = 13.91, *P* = 0.031; three-way interaction not significant) (marginal R^2^: 0.398, conditional R^2^: 0.529; Fig. 2A). Posthoc analyses demonstrated significant differences with short intense plots at both sites: fungal richness was significantly higher at the 11 month sampling period compared to previous sampling periods (2.06 fold increase at lower montane site and 1.50 fold increase at the upper montane site). In comparison, fungal richness did not differ among any other treatment pairs within site or over time (Fig. 2A).

**Figure 2.**
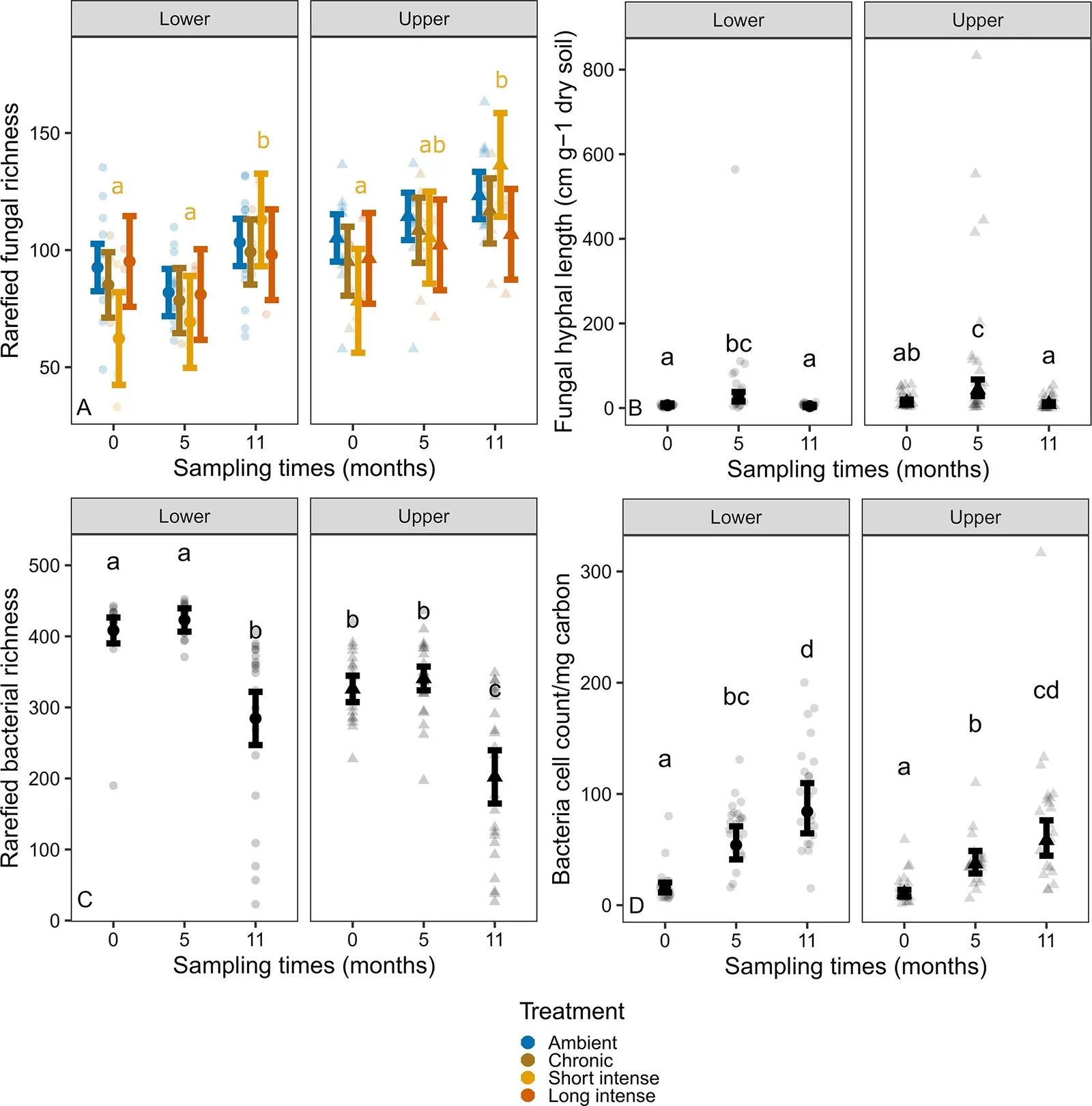
Estimated marginal means and 95% CI for (A) soil fungal rarefied richness, (B) soil fungal hyphal length, (C) soil bacterial rarefied richness, (D) soil bacterial cell counts (i.e. bacterial abundance) at a lower montane and upper montane site. Patterns of the four drought treatments—ambient (i.e. control), chronic (∼50% less precipitation for 11 months), short intense (∼100% less precipitation for 5 months), long intense (∼100% less precipitation for 11 months)—are only displayed when there was a significant effect. Note different letters indicate significant posthoc differences across all variables (only the short intense treatment showed a significant difference).

Fungal hyphal length (proxy for fungal abundance) significantly differed between sites (X^2^ (1) = 6.43, *P* = 0.011) and over time (X^2^ (2) = 51.67, *P* < 0.001; interactions were not significant) (marginal R^2^: 0.288, conditional R^2^: 0.341; Fig. 2B). Hyphal length did not significantly differ among drought treatments but was higher at the upper montane site. Abundance at both sites was highest at the 5 months sampling period (mid-June) with the abundance at the upper montane site 2.38 fold higher than the lower montane site.

The interactions between site and drought treatments (F_3,129_ = 1.62, *P* = 0.004, R^2^ = 0.026) and site and time (F_2,129_= 1.45, *P* < 0.001, R^2^ = 0.015) significantly influenced fungal community composition (Fig. S4A). Though very little variation explained these interactions (R^2^), the PCoA axis 1 explained 27.7% of the variation where there was greater site differentiation by 11 months (PCoA 1; Fig. S4A). In addition, communities from the chronic plots seem to separate from the other treatments in the upper montane site (Fig. S4A).

### Soil bacterial richness, abundance, and composition

Bacterial richness was not influenced by drought treatments but significantly differed over time (X^2^ (2) = 60.16, *P* < 0.001) and between sites (X^2^ (1) = 65.17, *P* < 0.001; interactions not significant; marginal R^2^: 0.719, conditional R^2^: 0.749; Fig. 2C). Bacterial richness was significantly higher (1.29 fold) at the lower montane site and declined at both sites over the sampling period between 5 and 11 months. Bacterial cell counts (i.e. abundance) were greater (1.4 fold) at the low elevation site (X^2^ (1) = 6.26, *P* = 0.012) and increased over time in both sites (X^2^ (2) = 179.83, *P* < 0.001; interactions not significant) (marginal R^2^: 0.551, conditional R^2^: 0.612; Fig. 2D). Drought treatments did not significantly affect bacterial abundance.

Interactions in site vs drought treatments (F_3,126_ = 1.58, *P* < 0.001, R^2^ = 0.023) and site vs time (F_2,126_ = 2.87, *P* = 0.003, R^2^ = 0.027) significantly influenced bacterial composition (Fig. S4B) with greater site differentiation by 11 months along the second axis (PCoA 2; Fig. S4B). Though very little variation explained these interactions (i.e. R^2^ values low), both PCoA axes explained at least 20% of the variation where there was greater site at the initital sampling period in mid- January (PCoA 1 (compared to PCoA 2 in fungi); Fig. S4B). There was less separation among drought treatments compared to fungal communities (Fig. S4B).

### Fungi functional groups

Saprotrophs were the only functional group significantly influenced by drought treatment (time and drought treatment interaction: X^2^ (6) = 13.49, *P* = 0.036; marginal R^2^: 0.616, conditional R^2^: 0.869; Fig. 3A); however, this seemed to be influenced by heterogeneity of plots at the initial sampling period in mid-January. Subsequent sampling periods showed no difference among drought plots. The proportion of endophytes were significantly higher at the lower site compared to the upper site (6.64 fold; X^2^ (1) = 42.55, *P* < 0.001; marginal R^2^: 0.355, conditional R^2^: 0.470; Fig. 3C), though at low proportions of reads (means < 0.007). In contrast, the proportion of symbionts (all types of mycorrhizal fungi) was significantly higher at the upper site than the lower site (3.6 fold; X^2^ (1) = 37.23, *P* < 0.001) and were not influenced by time or drought (*P* > 0.05; marginal R^2^: 0.376, conditional R^2^: 0.548; Fig. 3B). The proportion of plant pathogens was influenced by the time and site interaction (X^2^ (2) = 47.36, *P* < 0.001), but not drought (marginal R^2^: 0.253, conditional R^2^: 0.564; Fig. 3D). In the initial sampling period, pathogens (putative) were highest at the lower site at the initial sampling period but decreased over time; in comparison, pathogens were lowest at the upper site at the initial sampling period and increased over time (Fig. 3D; mean at lower site: 0.022 ± 0.003; mean and upper site: 0.019 ± 0.003).

**Figure 3.**
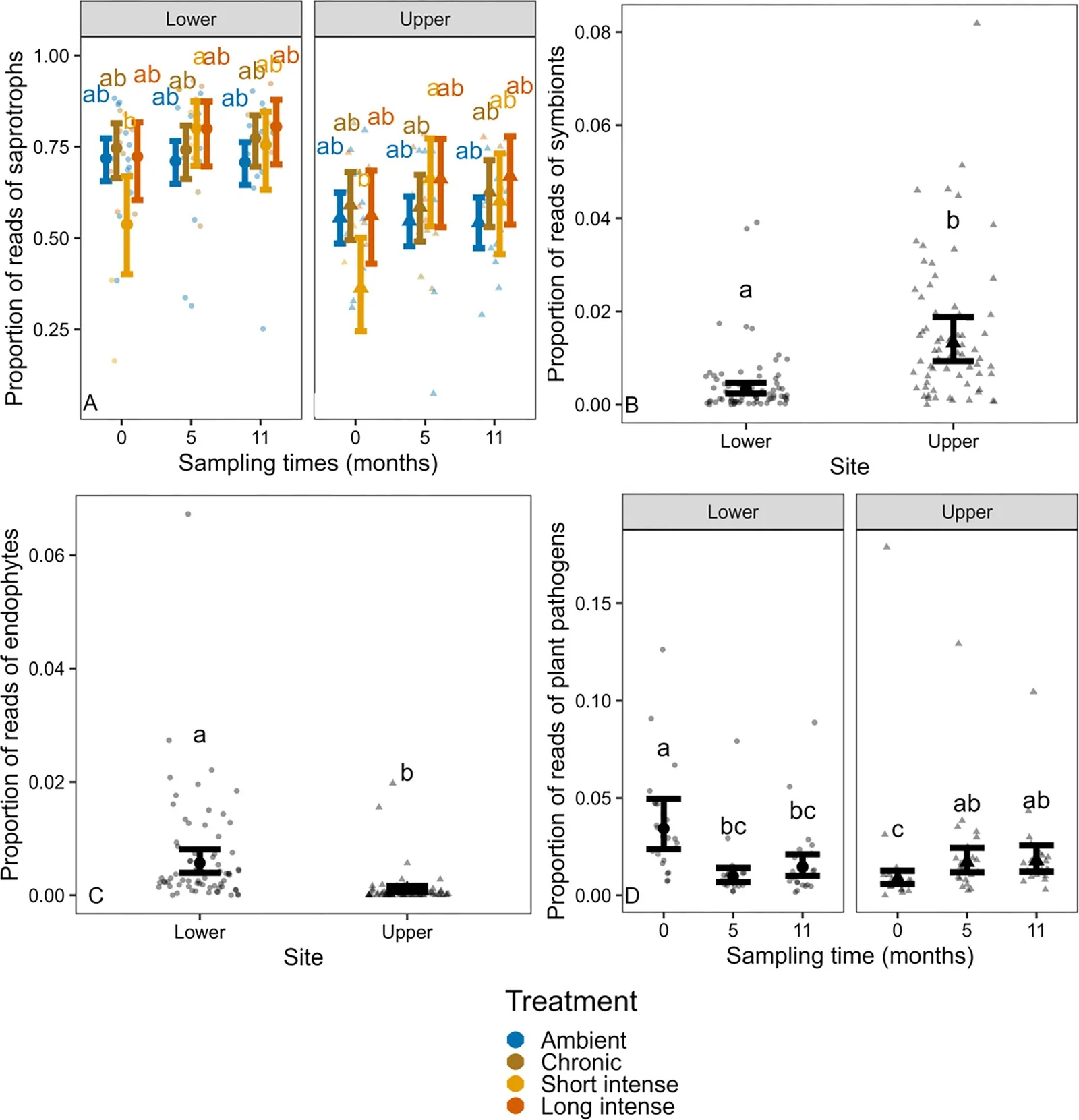
Proportion of fungal reads in a lower montane and upper montane site in functional guilds (A) saprotrophs, (B) symbionts, (C) endophytes and (D) putative plant pathogens. Patterns of the four drought treatments—ambient (i.e. control), chronic (∼50% less precipitation for 11 months), short intense (∼100% less precipitation for 5 months), long intense (∼100% less precipitation for 11 months)—are only displayed when there was a significant effect. Note different letters indicate significant posthoc differences across all variables.

### Microbial cage translocation

Fungal richness in the microbial cages significantly changed over time (X^2^ (1) = 5.32, *P* = 0.021; marginal R^2^: 0.164, conditional R^2^: 0.164; Fig. 4), but not between sites or among drought treatments. After 11 months, richness decreased 1.12 fold. Bacterial richness in cages did not differ between sites, time or treatments (*P* > 0.05).

**Figure 4.**
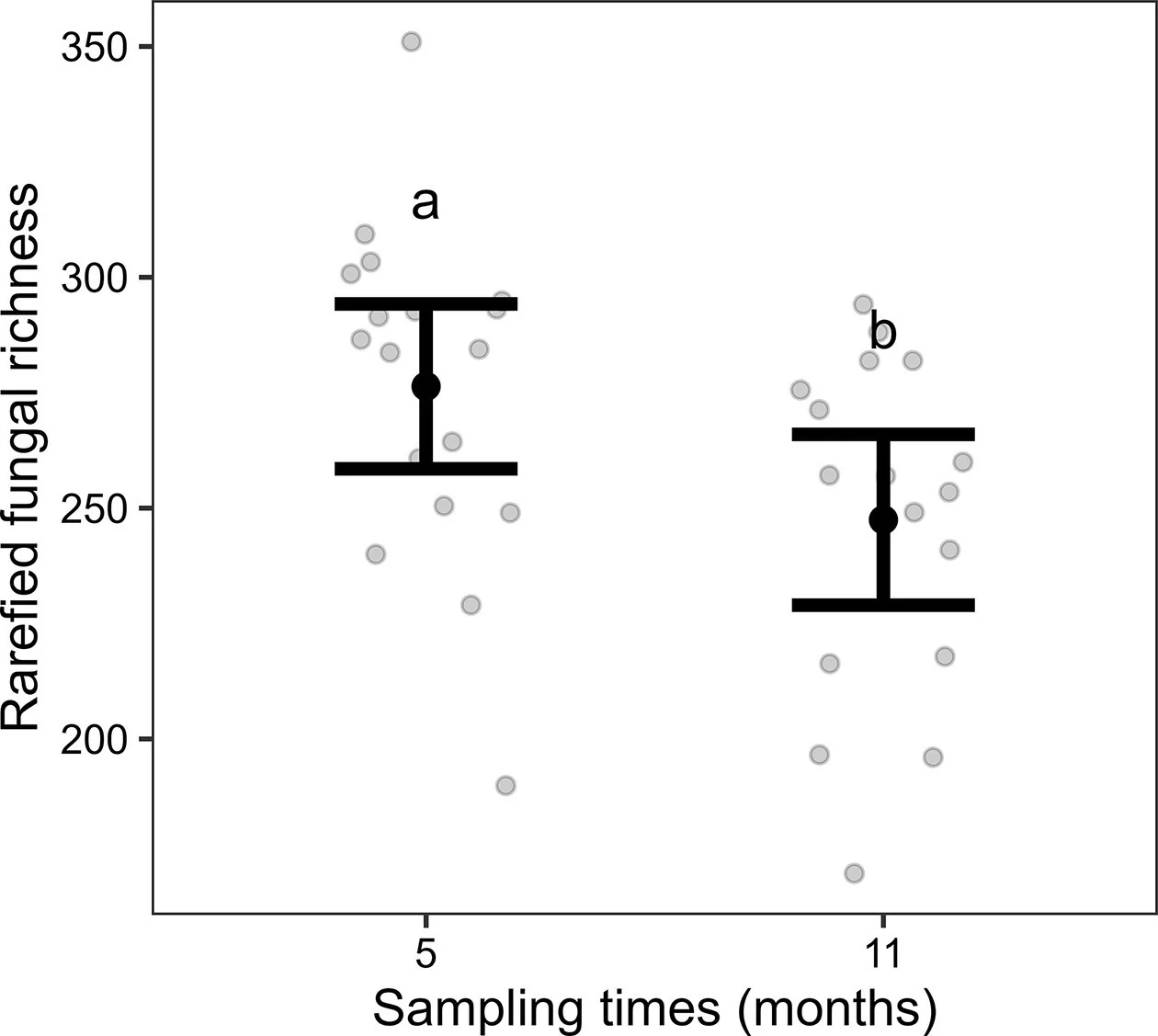
Estimated marginal mean soil fungal richness (± 95% CI) for a microbial translocation across time (cages were placed at sites at initial experimental set-up (0 months) and collected 5 and 11 months later). Note different letters indicate significant posthoc differences across all variables.

Only one two-way interactions significantly influenced fungal composition in cages (Fig. S5A): 1) drought effects depended on site (F_2,25_ = 1.21, *P* = 0.002, R^2^ = 0.062). The ambient treatment after 5 months separated out at the upper montane site but overlapped with the long intense after 11 months. The long intense separated out from the other treatments at both 5 months and 11 months at the lower site; all other treatments overlapped (Fig. S5A). Though R^2^ values were low PCoA 1 described 21.2% of the variation in fungal communities (Fig. S5A). A site and treatment interaction significantly influenced bacterial composition in cages (F_2,35_=0.98, *P* < 0.001, R^2^ = 0.043) and the main effect of time (F_1,35_ = 4.41, *P* < 0.001, R^2^ = 0.096; three-way interaction not significant) influenced community composition (Fig. S5B). By 11 months, treatments had separated more in comparison at 5 months (Fig. S5B). PCoA 1 described 28.8% of the variation in bacterial communities (Fig. S5B).

### Fungal functional groups

Differences among fungal functional groups were observed in the microbial cages. The three-way interaction among site, drought treatments and time was significant for saprotrophs (X^2^ (2) = 16.82, *P* < 0.001; marginal R^2^: 0.612, conditional R^2^: 0.970; Fig. 5A) and putative plant pathogens (X^2^ (2) = 27.03, *P* < 0.001; marginal R^2^: 0.384, conditional R^2^: 0.920; Fig. 5C). Specifically, the pairwise comparisons of the proportion of reads of saprotrophs did not differ within site and time periods but did differ between the sites (Fig. 5A). There was a greater increase in saprotrophs in the long intense plots in the upper montane site. The proportion of reads of plant pathogens declined in long intense from 5 to 11 months in the upper montane site but did not differ from ambient plots at each time point (Fig. 5C). Symbionts were influenced by the site and time interaction (X^2^ (1) = 9.22, *P* = 0.002; Fig. 5B) and the interaction between site and treatment (X^2^(2) = 8.53, *P* = 0.014; marginal R^2^: 0.519, conditional R^2^: 0.577). At the lower montane site, symbionts slightly increased under the long intense plot in comparison to decreasing at the upper montane site in the long intense plots. The proportion of reads of endophytes were influenced by site (X^2^(1) = 4.21, *P* = 0.040; Fig. 5D) and time (X^2^(1) = 4.49, *P* = 0.034; marginal R^2^: 0.411, conditional R^2^: 0.411). There was a slight increase in endophytes in the upper montane site compared to the lower montane site over time. However, posthoc pairwise comparisons did not indicate significant differences within sites over time.

**Figure 5.**
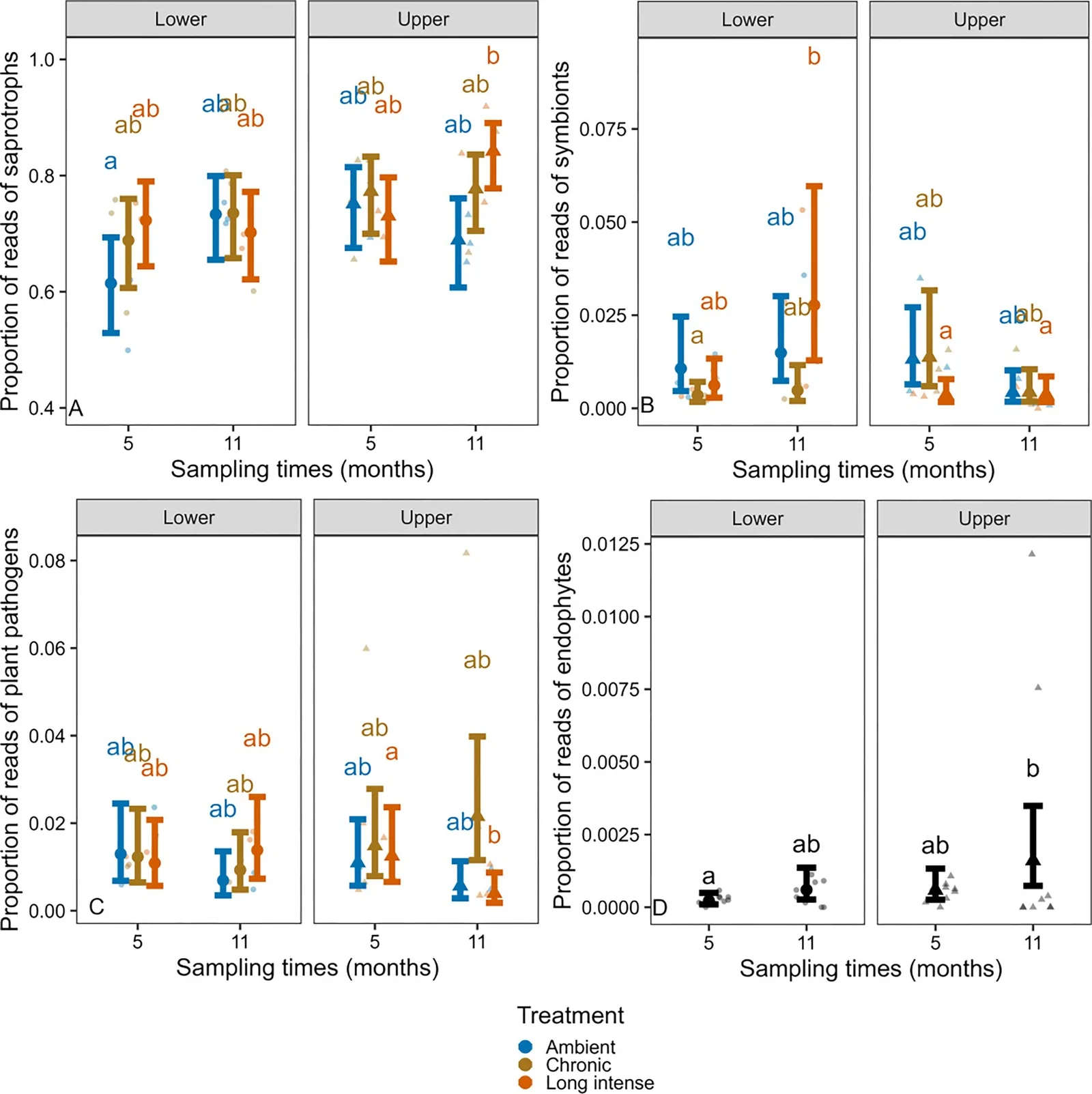
Estimated marginal mean (± 95% CI) for fungal functional groups as the proportion of fungal reads in a microbial translocation where (A) saprotrophs, (B) symbionts, (C) putative plant pathogens, and (D) endophytes. Soil from the upper montane site was translocated to the lower montane site (called lower in this figure) and control cages were placed at the upper montane site across three drought treatments: ambient (i.e. control), chronic (∼50% less precipitation for 11 months), and long intense (∼100% less precipitation for 11 months) and across time (cages were placed at sites at initial experimental set-up (0 months) and collected 5 and 11 months later). Patterns from the three drought treatments are only displayed when there was a significant effect. Note different letters indicate significant posthoc differences across all variables.

## Discussion

We examined how soil microbial communities are structured by elevation in a tropical montane forest, comparing a lower montane forest with an upper montane cloud forest, and how they respond to experimental warming and drought manipulations. The elevation difference between the sites—and the associated differences in climate—emerged as the dominant factor shaping microbial abundance, richness and community composition, as found in other tropical montane forests (Looby et al. 2016), often overriding the experimental drought treatments.

Both fungal richness and abundance trended higher in the upper montane cloud forest, suggesting that cooler, wetter conditions found at higher elevations in this study support fungal coexistence. This was opposite to what we hypothesised (hypothesis 1). It’s likely consistently high soil moisture at the upper montane site promotes hyphal persistence (Querejeta et al. 2012), while slower decomposition rates in cooler and wetter conditions favour the accumulation of organic matter and a diversity of complex carbon substrates (Salinas et al. 2011). Increased resource quantity and heterogeneity can expand niche space, allowing more fungal taxa to coexist while sustaining higher overall fungal abundance (Fierer et al. 2009).

In our study, drought treatments did not influence fungal abundance but we observed an overall decrease in fungal abundance in ambient conditions after 11 months, even as richness increased, which we surmise is a response to the general seasonal variation in precipitation in the site, especially the dry season (January through March) and peaks in rainfall in May and October (Martin and Fahey 2014). In contrast, the conditions in the lower montane site—higher temperatures, even greater seasonal variability in precipitation (Martin and Fahey 2014), and faster decomposition rates—reduce fungal richness, a pattern opposite to those seen at other lower elevation sites in other regions (Looby et al. 2016, Looby and Martin 2020). Together, these results indicate that cooler and wetter conditions in this system, rather than limiting diversity, promote structurally and functionally complex fungal communities.

Patterns in bacterial richness and abundance were the opposite of fungi, as both were higher at the lower montane site and confirmed our hypothesis that richness patterns between fungi and bacteria behave differently. The relative abundance of fungi and bacteria (i.e. fungi: bacteria) tend to be higher in soils with a low pH or a high C: N (Rousk et al. 2010, Waring et al. 2013), and indeed the soils in upper montane site in our study had a lower pH and higher C and N levels than the lower montane site. The upper montane site had slower decomposition rates of a uniform substrate in our experiment, a result consistent with a fungal-dominated, slow-cycling system (Sundqvist et al. 2013).

Functional guild patterns support this elevational difference. Saprotrophs were more abundant at the lower montane site and increased slightly under drought treatments, aligning with the faster decomposition at lower elevation and under reduced moisture (Looby and Treseder 2018). In contrast, symbiotic fungi were more prevalent at the upper elevation and were largely insensitive to drought, suggesting this functional group could be outcompeting saprotrophs for carbon (Gadgil and Gadgil 1971, 1975, Looby et al. 2016).

Decomposition patterns in our study were strongly influenced by conditions in the sites and treatments with several clear trends: 1) decomposition was unmistakably faster at the lower elevation; 2) decomposition was markedly slower in the drought treatments, independently of elevation, and stronger drought treatments invariably resulted in slower decomposition; and 3) the effect of 100% drought (i.e. the ‘short intense’ and the ‘long intense’ treatments), in slowing decomposition was much greater at higher elevation. Other studies have found that experimental drought led to slower mass loss rates (Santonja et al. 2015). A large experiment across the tropics in montane forest compared to lowland forests also found that climate was the predominant control on decomposition rates (Ostertag et al. 2022), with rates significantly slower in high elevation sites. We expected the wet, cloudy conditions in the higher elevation to limit decomposition rates (e.g. due to anoxic conditions), as hypothesized and documented in other tropical montane cloud forests (Bruijnzeel 2005), and so expected drought might increase decomposition rates by lowering moisture in the forest floor, but this was not the case (hypothesis 3 disproved), perhaps because our upper elevation site was not as anoxic as we expected.

Microbial communities were not necessarily more sensitive to drought and warming at the upper montane site. Drought increased fungal richness at both elevations and more so in the short intense treatment. Bacterial richness did not respond to drought (hypothesis 2 and 4 disproved). Bacteria may be less sensitive to drought due to their high turnover rates (bacteria biomass turns over 50% faster than fungal biomass; Waring et al. 2013) or better adapted to droughts (Schimel et al. 2007), which occur regularly in this region during the El Niño phase of the El Niño -Southern Oscillation (ENSO) cycle (Martin and Fahey 2006, Swann et al. 2023). Other research has demonstrated that fungi tend to be less resistant to drought than bacteria, and that fungal recovery to extreme events is slower (De Vries et al. 2018, Hannula and Veen 2025). Despite a demographic resistance, bacterial community composition was altered by drought, indicating compositional turnover can occur without changes in bulk richness or abundance (Allison and Martiny 2008).

The soil translocation experiment also did not indicate clearly that increases in temperature (combined with decreases in precipitation) would further influence soil fungal communities. Saprotrophs in the microbial cage translocation experiment did increase under drought in the upper montane site but those translocated to the low montane site seemed to remain stable. This suggests that some functional groups at the upper montane site have some resistance to drought under warmer conditions. Recall that only soil from the upper montane site was caged. Functional guild responses further demonstrated community reorganization following translocation and drought. Specifically, plant pathogens at the upper montane site decreased under long intense treatments. Together, these results indicate that upper montane fungal communities are diverse and may not be as vulnerable to climatic perturbation as hypothesized.

## Conclusion

Our results provide partial support for our initial hypothesis that higher elevation ecosystems support lower microbial richness, as fungal richness was slightly higher at the upper montane site, while bacteria richness was markedly lower at the upper montane site. We also hypothesised greater sensitivity of upper montane microbial communities to environmental change, which our results did not support: while fungal richness and composition were responsive to drought and translocation, bacterial richness and abundance were largely insensitive, indicating that sensitivity is microbial-group specific.

As climate change alters temperature and precipitation regimes in tropical mountains, shifts in microbial community composition, particularly among fungal functional groups, are likely to occur; however, these microbial communities may be more resistant to climate change than originally thought. It is possible that the 11-month duration of our study and focus on a single site which experiences an annual dry season and regular ENSO-triggered droughts do not capture the potential sensitivity of the microbial community to climate change in other tropical montane forests. Further understanding the impacts of climate change on microbial community dynamics will be critical for forecasting carbon storage and nutrient cycling in tropical montane forests under a changing climate.

## Supplementary Material

fiag112_Supplemental_File
