# Supplementary material for: Ambient climate and experimental drought strongly shape the richness and abundance of soil microbial communities in a tropical montane cloud forest
Source: FEMS Microbiol Ecol. 2026 Oct 12;102(11):fiag112. doi: 10.1093/femsec/fiag112 (PMC13673987; doi:10.1093/femsec/fiag112)
Supplement: Supplementary file 1 [file fiag112_supplemental_file.docx]

**Supplementary Materials for**

**“**Ambient climate and experimental drought strongly shape the richness and abundance of soil microbial communities in a tropical montane cloud forest”

Sarah J. Sapsford^1^, Patrick H. Martin^2^

^1^ Harry Butler Institute, Murdoch University, 90 South Street, Murdoch, 6150, Western Australia, Australia

^2^ Department of Biological Sciences, University of Denver, 2190 E. Iliff Avenue, Denver, CO 80208 USA

|   **A** |   **B** |
| --- | --- |

**Figure S1**: Drought shelters (1-m^2^ in area, mounted 1-m above the forest floor) in the upper montane site (2250 m a.s.l.) in tropical montane cloud forest on the windward slopes of the Cordillera Central in Armando Bermudez National Park, Dominican Republic. Panel (A) displays a ‘chronic’ treatment shelter with 50% open area; panel (B) is an ‘intense’ treatment shelter with 0% open area.

**Figure S2:** Daily mean soil temperature from datalogging Decagon 5TM soil probes, recorded every 15 minutes through the duration of the study. Probes were placed horizontally 7.5 cm below the soil surface in the center of a plot. Six 5TM probes were placed in each site, but several dataloggers failed (e.g. water intrusion into dataloggers, battery failure) either during or for the duration of the study.

**Figure S3:** Soil moisture (volumetric water content, %) from datalogging 5TM and EC-5 Decagon soil probes, recorded every 15 minutes through the duration of the study. Probes were placed horizontally 7.5 cm below the soil surface in the center of a plot. Twelve probes were placed in each site, but several dataloggers failed (e.g. water intrusion into dataloggers, battery failure) either during or for the duration of the study.

**Figure S4:** Principal coordinates analysis of A) soil fungal and B) soil bacterial communities at a lower montane and upper montane site across four drought treatments: ambient (i.e. control), chronic (~50% less precipitation for 11 months), short intense (~100% less precipitation for 5 months) and long intense (~100% less precipitation for 11 months) and across time (0 months,5 months, and 11 months post-treatment). PCoA scores for each ordination axis and site/treatment group were averaged (±1SEM) into centroids.

**Figure S5:** Principal coordinates analysis of A) soil fungal communities and B) soil bacterial communities in a microbial cage translocation. PCoA scores for each ordination axis and site/treatment group were averaged (±1SEM) into centroids. Soil from the upper montane site was caged and translocated to the lower montane site; control cages were also placed in the upper site. Cages were placed in drought treatments in both sites: ambient (i.e. control), chronic (50% less precipitation for 11 months) and long intense (100% less precipitation for 11 months). Cages were deployed in January (0 months), and half were collected after 5 months and half after 11 months.
